# Peculiarities of cardio-respiratory relationships in qualified athletes with different types of heart rhythm regulation according to respiratory maneuver data

**DOI:** 10.3389/fspor.2024.1451643

**Published:** 2025-01-13

**Authors:** Oleksandr Romanchuk

**Affiliations:** ^1^ Department of Internal and Family Medicine, Lesya Ukrainka Volyn National University, Lutsk, Ukraine; ^2^ Department of Therapy and Rehabilitation, Ivan Boberskij Ivan Bobersky Lviv State University of Physical Culture, Lviv, Ukraine

**Keywords:** heart rhythm regulation, athletes, respiratory maneuver, arterial baroreflex sensitivity, hildebrandt index, volume synchronization index

## Abstract

**Introduction:**

Our goal was to determine the differences in changes in cardiovascular and cardiorespiratory interaction indicators during a respiratory maneuver with a change in breathing rate in athletes with different types of heart rate regulation.

**Methods:**

The results of a study of 183 healthy men aged 21.2 ± 2.3 years, who were systematically involved in various sports, were analyzed. According to the results of the analysis of the HRV study during spontaneous breathing, the athletes were divided into 4 groups taking into account the type of heart rate regulation (HRR). Group 1 (with type I) consisted of 53 people, group 2 (with type II)—29 people, group 3 (with type III)—85 people, group 4 (with type IV)—16 people. The methodology for studying the cardiorespiratory system included combined measurements of the respiratory and cardiovascular system activity indicators in a sitting position using a spiroarteriocardiorhythmograph. The duration of the study was 6 min.

**Results:**

According to changes in cardiorespiratory and cardiovascular interaction indicators during controlled breathing with a frequency of 6 and 15 per minute (CR_6_ and CR_15_), it is shown that with a pronounced predominance of parasympathetic influences (type IV) in conditions of excessive cardiorespiratory control and moderate hyperventilation, differences in changes in arterial baroreflex sensitivity (δBR_LF_ and δBR_HF_) are noted in comparison with other HRR. Athletes with type IV at CR_6_ in δBR_LF_ significantly differ from athletes with type III (*p* = 0.026) and do not differ from athletes with type II (*p* = 0.141). In δBR_HF_ significantly (*p* = 0.038 and *p* = 0.043)—from athletes with types I and II. It is shown that with the predominance of sympathetic influences (types I and II), the reactivity of BRS (δBRLF and δBR_HF_) in response to moderate hyperventilation (CR_15_) is significantly lower. Changes in the Hildebrandt index and the volume synchronization index additionally differentiate HRR associated with a moderate and pronounced predominance of sympathetic and parasympathetic influences.

**Conclusion:**

The use of a respiratory maneuver in a combined study of the cardiorespiratory system in the conditions of current control of athletes showed informativeness in the differentiation of HRR types and states of functional overstrain.

## 1 Introduction

The problem of rapid evaluation of the functional state of the athletes' body is extremely relevant in the conditions of training and competitions (1, 2). First of all, this is due to the need to objectify changes in the body as soon as possible (3, 4). Most often, for this purpose, a number of biochemical and instrumental studies are used, which allow assessing the impact of physical exertion, the body's reaction and the course of recovery processes (5–11). Among the instrumental research methods are the analysis of electrical activity of the heart, heart rate variability (HRV), sensorimotor function, and others (12–18).

The heart, as an indicator of the adaptive reactions of the entire organism, reacts to a wide variety of internal and external influences, which is reflected in the indicators of HRV. HRV indicators provide important information about the state of the autonomic nervous system (ANS) and other levels of neurohumoral regulation, therefore research and analysis of HRV is considered a methodology for studying the mechanisms of regulation of physiological functions in the human body (19–26). Taking into account the results of the HRV study allows to increase the effectiveness of diagnosing the condition of athletes, to preventing fatigue, adjusting the training load and assessing the athlete's ability to self-regulate (27–30). The main advantage of HRV is that it is non-invasive, cheap, time-efficient and can be used regularly and simultaneously in a large number of athletes (20, 31–33). According to the two-circuit model of heart rhythm control, which is based on the regulatory activity of the sympathetic and parasympathetic divisions of the ANS Shlyk et al. (34) proposed a .classification of heart rhythm regulation (HRR) types, which provides for the selection of moderate and pronounced variants of the predominance of central (types I and II) and autonomous (types III and IV) regulation circuits. This classification takes into account the values of indicators of total HRV power—TР (ms^2^), variability in a very low range—VLF (ms^2^) and stress index—SI (с.u.). The feasibility of using these indicators to assess the current functional state of regulatory systems in healthy people and athletes has been shown in a number of other studies (5, 24, 29, 35, 36). Each of the types of HRR is characterized by the presence of possible states at rest. At the same time, a number of studies showed the individual typological features of such a classification, when a certain type of regulation in an individual person in a state of rest is preserved for a long time, and its changes are caused by the modification of external and internal influencing factors (37). The technique is widely used during monitoring in the educational and training process of athletes. It was even mentioned in the instructions for monitoring in national teams of different countries. Its results make it possible to predict the athlete's functional readiness and the possibility of achieving a sports result (37, 38).

The search for informative criteria for assessing the functional state of athletes' bodies during express screening examinations within the educational and training process arouses interest in the analysis of combined intersystem changes in the cardiorespiratory system (39–44). Such an opportunity is available with the simultaneous registration of indicators of cardiovascular and respiratory systems, because it allows direct assessment of the mechanisms of intersystem interaction (21, 45–53). This is possible under the condition of simultaneous registration of cardio intervals, pulse wave of blood pressure and respiratory flows, which is achieved with the help of Finapres (54) and SAСR (55) devices.

Studying indicators of intersystem interaction, especially taking into account the frequency and depth of breathing, both spontaneous and controlled, is important in understanding HRV changes in the regulation of the autonomic nervous system (49, 50, 56–61). In earlier studies, it was shown that in the range from 6 to 10 spontaneous breaths per minute, there is a clear relationship between the frequency of breathing and indicators of HRV (26, 62). Previous studies have shown that the alternate execution of tests with controlled breathing with a frequency of 0.1 Hz and 0.25 Hz allows determining the peculiarities of the ANS response, which relate to multidirectional changes in most indicators of HRV, blood pressure variability, partially hemodynamics and indicators of the cardiorespiratory system interaction. An assumption was made about the probable prognostic value of these changes in determining the functional state of the athletes' body and patients with different diseases (63–69). A number of authors have proposed assessing the redundancy of cardiorespiratory control and the synergy of this connection during breathing at a rate lower than spontaneous (70).

Among the known indicators of cardiorespiratory interaction—the indicators of the arterial baroreflex sensitivity (BRS) in the low-frequency (BR_LF_, ms × mmHg^−1^) and high-frequency (BR_HF_, ms × mmHg^−1^) ranges, which characterize the interaction of the cardiac and vascular components of hemodynamic support (71), and also the indicators of frequency (Hildebrandt index, c.u.) and volumetric (VSI, dm^3^ × L^−1^) synchronization of the heart and breathing, which are more related with the oxygenation capabilities of the body (72, 73). Their changes during controlled breathing have not been studied enough. In our opinion, their research and analysis can in the future significantly supplement the possibilities of current monitoring of the functional state of athletes.

In this paper, our goal was to determine the differences in the changes in indicators of cardiovascular and cardiorespiratory interaction during a breathing maneuver with a change in breathing frequency in athletes with different types of heart rhythm regulation.

## 2 Materials and methods

### 2.1 Study subjects

This study was conducted in the limits scientific programs of departments of Exercises Medicine and Sports Medicine of South Ukrainian National Pedagogical University (September 2012–July 2016) and Sports Medicine of Lviv State University of Physical Culture (January 2021), on different sports bases of Odesa, Lviv and of team Ukraine. We analyzed the results of the study of 183 healthy men aged 21 ± 2 years who regularly engaged in various sports, did not complain of any problems in the state of the body, did not have acute diseases and were allowed to participate in sports according to the results of the last medical examination. The length of time in sports ranged from 3 to 15 years, the level of sportsmanship ranged from a candidate for master of sports to champions of Ukraine, Europe, the World, and the Olympic Games. All examinations were carried out in the morning, 2–3 h after a light breakfast. On the eve of the study, all participants were instructed by trainers to avoid consumption of stimulant beverages (coffee, green tea, energy drinks) before the examination. Taking into account that the examination was carried out in different periods of the annual training cycle, the main condition for admission to the study was the absence of intense and prolonged physical load the day before. Among the athletes who were examined were representatives of athletics (long and middle distance runners), rowers on kayaks and canoes, table tennis players, representatives of single combat sports (boxing, karate, freestyle wrestling, judo, Greco-Roman wrestling), representatives of games (volleyball, water polo, handball, soccer), as well as gymnasts, acrobats and shooters.

### 2.2 Procedure of study

The procedure for studying the cardiorespiratory system included conducting combined measurements of indicators of activity of the respiratory and cardiovascular systems in a sitting position using a Spiroarteriocardiorhythmograph (SACR) device (RRID:SCR_025431). The duration of the study was 6 min and involved the sequential registration of three measurements (2 min each) with a change in breathing rate. During the first 2 min, SACR indicators were recorded during normal spontaneous breathing (SR), during the second 2 min—during controlled breathing 6 times per minute (5 s inhalation, 5 s exhalation) (CR_6_), during the third 2 min—during controlled breathing 15 once per minute (2 s inhalation, 2 s exhalation) (CR_15_).

**Figure 1:**
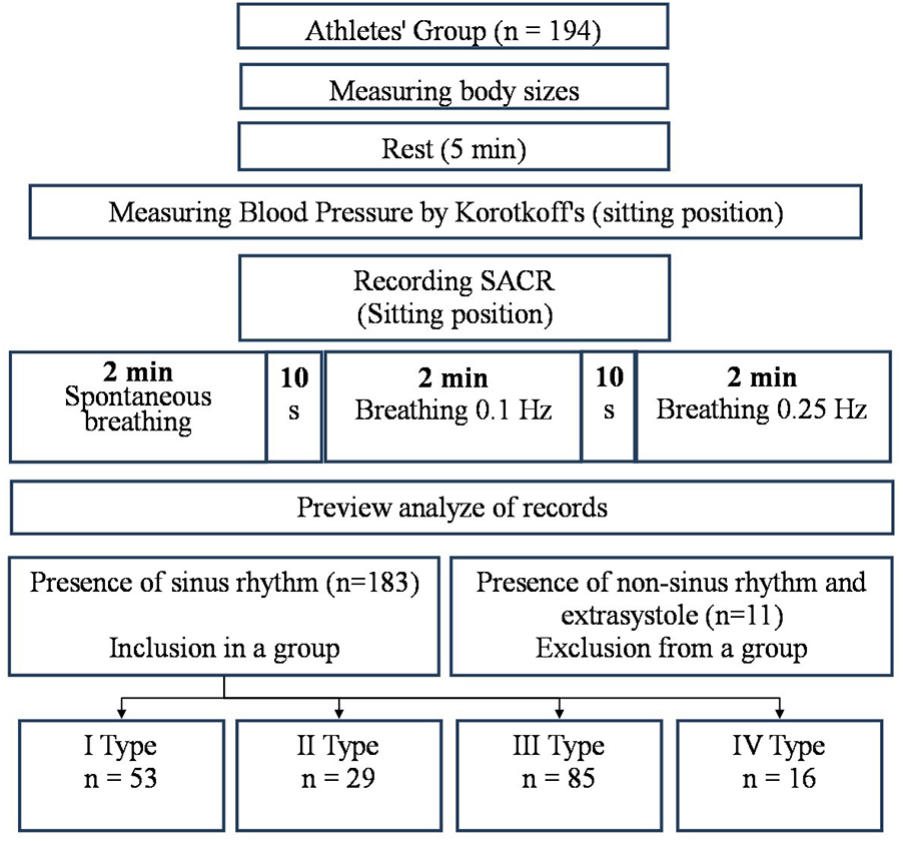
Design of research.

The only condition for exclusion from the analysis of the examination results was the presence of heart rhythm disturbances in the form of extrasystoles and non-sinus rhythm, which was establishing during the preliminary analysis of the examination records. A total of 11 such cases were registered. From 11 mentioned cases there were extrasystoles in three cases and non-sinus rhythm in two cases in rest. In six cases the extrasystoles appeared in breathing 6 times per minute. They are not included in the analysis group (Figure 1). There were no other complaints or violations during and after the breathing maneuver.

This study was approved by the Ethics Committee of the South Ukrainian National Pedagogical University (No. 121), by the Ethics Committee of the Lviv State University of Physical Culture (No. 33–16). All athletes were informed about the study and signed an informed consent form before the trial.

### 2.3 Method

The study was conducted using the SAСR device. ECG recording in 1 lead allowed to determine the indicators of HRV according to the spectral analysis of the sequence of RR intervals is total power (ТР, ms^2^), power in the very low frequency range (VLF, ms^2^), power in the low frequency (LF, ms^2^) and power in the high frequency range (HF, ms^2^) and their derivatives (LFn, n.u., HFn, n.u., LF/HF); according to the math analysis of the sequence of RR intervals were defined RMSSD (square root of the sum of squares of the differences in the values of consecutive pairs of normal intervals, ms), pNN50 (the percentage of NN50 from the total number of consecutive pairs of intervals that differ by more than 50 milliseconds, obtained over the entire time recording,%) (74); according to cardiointervalometry were defined the heart rate (HR, min^−1^), averages durations and intervals of PQRST-complex and indicator of systemic hemodynamics (75)—cardiac output (CO, dm^3^); according to the pulse wave recording with the help of a photoplethysmographic sensor on the finger by the Penaz method (54, 76), blood pressure variability in ranges similarly to HRV were determined a total power of SBP variability and DBP variability (ТР_SBP_, mmHg^2^ and ТР_DBP_, mmHg^2^), power in the very low-frequency range (VLF_SBP_, mmHg^2^ and VLF_DBP_, mmHg^2^), power in the low-frequency range (LF_SBP_, mmHg^2^ and LF_DBP_, mmHg^2^) and power in the high-frequency range (HF_SBP_, mmHg^2^ and HF_DBP_, mmHg^2^) (77–79). By using the spectral method we determined the index of arterial baroreflex sensitivity (BRS, ms × mmHg^−1^)—*α*-coefficient, that was calculated in high (BR_HF_) and low (BR_LF_) frequencies ranges (80–82).

$$BR_{\mathrm{LF}}=\sqrt{LF_{\mathrm{HRV}\;}/LF_{\mathrm{SBP}}}$$

$$BR_{\mathrm{HF}}=\sqrt{HF_{\mathrm{HRV}\;}/HF_{\mathrm{SBP}}}$$

The ultrasonic sensor of the SACR device allows to measure flows of air on inspiration and expiration and to define the average parameters of a respiration: duration of inspiratory (T_I_, s), duration of expiratory (T_E_, s), respiratory rate (RR, min^−1^) tidal volume (V_T_, L), as well as the volume of minute respiration (V_E_, L × min^−1^) (83, 84).

Indicators of frequency and volume synchronization of the cardio-respiratory system were also calculated—Hildebrandt index (HI) and VSI (72, 85).

$$\mathrm{HI}=\mathrm{HR}\;(\mathrm{mi}n^{-1})/\mathrm{RR}(\mathrm{mi}n^{-1})$$

$$\mathrm{VSI}=\mathrm{CO}\;(dm^{3}\times \mathrm{mi}n^{-1})/V_{E}\;(L\times \mathrm{mi}n^{-1})$$

The type of HRR was studied in all athletes and was estimated in accordance with the method proposed by Shlyk et al. (34) (Table 1). Type III is considered the most optimal, which characterizes a moderate predominance of parasympathetic effects, which is characteristic of most people. Types I and II testify to the predominance of the central link activity of regulation and the development of moderate and severe sympathicotonia, but also reflect the activation of stress-realizing systems in the body. As for type IV, when there is a high variability of the heart rate, such a predominance of parasympathetic influences, on the one hand, may indicate the imperfection of central regulation and the development of autonomic dysfunctions, and on the other hand, the peculiarities of autonomic regulation during systematic training loads, especially during the development of the supercompensation condition in athletes (37, 38). In general, II and IV types of HRR are considered unstable and may characterize autonomic dysregulation.

**Table 1:** Criteria for determining types of heart rhythm regulation according to N.I. Shlyk.

Types of heart rhythm regulation	Type	Criteria	
		SI (c.u.)	VLF (ms^2^)
Predominance of central regulation	І	>100	>240
	ІІ	>100	<240
Predominance of autonomous regulation	ІІІ	30–100	>240
	IV	<30	VLF > 500, TP > 8000–10000

Abbreviations: SI, stress index; VLF, very low frequency; TP, total power.

The principles of classification of HRR types, taking into account the above criteria, are presented in Table 1.

According to the analysis of the HRV study results during spontaneous breathing, the athletes were divided into 4 groups, taking into account the type of HRR. Group 1 (with type I) consisted of 53 people, group 2 (with type II)—29 people, group 3 (with type III)—85 people, group 4 (with type IV)—16 people.

### 2.4 Statistical analysis

The results were processed using the STATISTICA program for Windows (version 10.0), Microsoft Excel 2012. The obtained data are presented as medians with 25%–75% (Q1; Q3) percentiles. Differences between initial and subsequent measurements were obtained using the Wilcoxon matched pairs test. At the next Two Way ANOVA test was performed.

## 3 Results

### 3.1 Morphofunctional data result

In the Table 2 presents the differences in the main morphometric indicators of athletes of the studied groups.

**Table 2:** Morphometric characteristics of athletes with different types of heart rhythm regulation.

Indicator	Values			
	Type I *n* = 53	Type II *n* = 29	Type III *n* = 85	Type IV *n* = 16
Age, y (m ± SD)	21.2 ± 2.3	20.3 ± 2.8	21.1 ± 2.4	20.9 ± 1.5
Weight, kg	75.0 (69.0; 82.0)	74.5 (70.0; 80.0)	73.0 (68.0; 80.0)	67.0 (65.0; 72.0)
Height, cm	178.0 (175.0; 182.0)	178.0 (173.5; 183.0)	177.0 (173.0; 181.0)	176.5 (173.5; 178.0)
BMI, kg × m^−2^	23.5 (21.8; 25.4)	23.5 (22.3; 24.5)	23.5 (22.2; 24.6)	22.1 (20.7; 24.1)
Fat, %	14.5 (11.4; 18.2)	15.1 (13.3; 16.8)	14.1 (10.8; 17.1)	15.1 (12.3; 17.3)

Abbreviations: BMI, body mass index.

Analyzing the morphometric indicators, it should be noted that probable differences were obtained for the body weight of athletes with the IV type of HRR. Their weight was significantly lower compared to athletes of other groups (types I–III) (*р* = 0.010; *р* = 0.044; *р* = 0.027, respectively). The height of athletes of types I and IV also differed, *p* = 0.024, which indicated its lower values in athletes with an excessive predominance of parasympathetic regulation.

### 3.2 Intergroup differences of the main indicators used for the analysis of changes during a breathing maneuver at rest

According to the proposed classification (34, 37), groups of athletes were quite clearly differentiated by HRV indicators—TP (ms^2^), VLF (ms^2^), SI (c.u.), which was quite expected (Table 3). As for other indicators of HRV, it should be noted that differentiation by indicators characterizing the activity of the parasympathetic link of the ANS—HF (ms^2^), RMSSD (ms) and pNN50 (%), for groups with a predominance of sympathetic influences (I and II types) not noted, *p* = 0.226; *p* = 0.096 and *p* = 0.873, respectively. Also, HR (min^−1^) did not differ between athletes with type I and type II. Most notable is the fact that none of the groups differed in LF/HF (ms^2^/ms^2^), which is most often used to characterize sympathy-vagal balance. Informative was the absence of differences between indicators of SBP variability in the low-frequency (SBP_LF_, mmHg^2^) and high-frequency (SBP_HF_, mmHg^2^) ranges, which were later used to calculate BR_LF_ (ms × mmHg^−1^) and BR_HF_ (ms × mmHg^−1^). That is, differences in BRS indicators (BR_LF_ and BR_HF_) at rest were determined by differences in heart rate power in LF and HF ranges (LF, ms^2^ and HF, ms^2^). This allows us to state that the proposed approach to assessing HRR types is clearly combined with the differences in BRS in the low-frequency and high-frequency ranges. The highest BRS values are for type IV, slightly lower for type III, even lower for type I, and the lowest for type II of HRR. Accordingly, a significant predominance of parasympathetic influences is characterized by the largest BRS, and an excessive predominance of sympathetic ones is characterized by the smallest.

**Table 3:** Intergroup differences of the studied parameters, Med (Q1;Q3), *n* = 183.

Indicator	Values				*p*					
	Type I *n* = 53	Type II *n* = 29	Type III *n* = 85	Type IV *n* = 16	I–II	I–III	I–IV	II–III	II–IV	III–IV
Age, y (m ± SD)	21.2 ± 2.3	20.3 ± 2.8	21.1 ± 2.4	20.9 ± 1.5						
HR, min^−1^	74.1 (65.1; 80.8)	74.8 (65.4; 81.2)	65.1 (61.4; 70.8)	62.2 (57.5; 66.5)	0.701	**0** **.** **000**	**0**.**000**	**0**.**004**	**0**.**000**	**0**.**012**
SBP, mmHg	114 (110; 126)	118 (110; 124)	114 (104; 122)	110 (104; 122)	0.321	**0**.**042**	**0**.**033**	**0**.**048**	**0**.**038**	0.240
DBP, mmHg	74 (64; 86)	76 (68; 86)	70 (60; 80)	68 (60; 76)	0.654	**0**.**034**	**0**.**026**	**0**.**041**	**0**.**014**	0.346
**TP, ms^2^**	**2,704** (**2285; 4651)**	**1,823** (**1475; 2652)**	**5,314** (**3881; 7674)**	**13,833** (**10491; 18376)**	**0**.**000**	**0**.**000**	**0**.**000**	**0**.**000**	**0**.**000**	**0**.**000**
**VLF, ms^2^**	**581** (**335; 894)**	**169** (**104; 207)**	**538** (**286; 882)**	**1,024** (**764; 1513)**	**0**.**000**	**0**.**000**	**0**.**000**	**0**.**000**	**0**.**000**	**0**.**000**
LF, ms^2^	924 (557; 1892)	557 (331; 824)	1,640 (1018; 3352)	4,487 (3177; 9220)	**0**.**000**	**0**.**000**	**0**.**001**	**0**.**000**	**0**.**000**	**0**.**038**
HF, ms^2^	1,089 (661; 1665)	1,129 (600; 1756)	2,642 (1282; 3931)	5,789 (2942; 11182)	0.226	**0**.**000**	**0**.**001**	**0**.**000**	**0**.**001**	**0**.**016**
LFHF, ms^2^/ms^2^	0.81 (0.49; 1.69)	0.49 (0.25; 1.21)	0.81 (0.25; 1.69)	1.00 (0.43; 1.69)	0.873	0.439	0.870	0.654	0.984	0.595
**SI, c.u.**	**172.1** (**129.3; 232.3)**	**210.0** (**136.6; 362.4)**	**59.7** (**45.0; 75.3)**	**24.9** (**16.6; 28.7)**	**0**.**000**	**0**.**000**	**0**.**000**	**0**.**000**	**0**.**000**	**0**.**000**
LF_SBP_, mmHg^2^	6.8 (4.0; 13.0)	7.3 (4.8; 9.6)	6.3 (3.2; 9.6)	9.0 (6.3; 13.0)	0.282	0.764	0.982	0.310	0.280	0.777
HF_SBP_, mmHg^2^	5.3 (2.9; 9.0)	5.3 (3.6; 12.3)	4.8 (2.3; 9.0)	4.9 (2.9; 12.3)	0.805	0.613	0.887	0.376	0.967	0.598
CO, dm^3^	4.8 (4.3; 5.4)	4.7 (4.3; 4.9)	4.4 (4.0; 4.8)	4.3 (4.0; 4.7)	0.201	**0**.**009**	**0**.**007**	0.464	0.159	0.324
RR, min^−1^	15.7 (13.2; 17.9)	14.7 (12.7; 16.7)	13.4 (11.0; 16.5)	12.9 (9.8; 15.4)	0.625	**0**.**001**	**0**.**033**	**0**.**014**	0.070	0.665
V_E_, L × min^−1^	8.5 (6.3; 10.5)	8.6 (7.0; 9.7)	7.6 (6.3; 8.9)	7.1 (5.1; 9.3)	0.857	0.942	0.092	0.859	0.143	0.240
RMSSD, ms	33.6 (27.9; 41.9)	31.8 (19.9; 37.5)	57.7 (46.7; 74.0)	110.8 (86.8; 141.1)	0.096	**0**.**000**	**0**.**000**	**0**.**000**	**0**.**000**	**0**.**001**
pNN50,%	11.11 (10.10; 12.66)	11.11 (10.10; 12.82)	13.16 (11.90; 18.35)	27.31 (14.09; 37.77)	0.873	**0**.**000**	**0**.**000**	**0**.**000**	**0**.**000**	**0**.**002**
VSI, dm^3^ × L^−1^	0.548 (0.425; 0.821)	0.572 (0.443; 0.687)	0.591 (0.510; 0.723)	0.648 (0.490; 0.885)	0.321	0.403	0.844	0.698	0.437	0.558
Hildebrandt index, c.u.	4.52 (3.98; 5.82)	4.85 (4.40; 5.72)	4.82 (3.98; 6.36)	5.05 (3.85; 6.57)	0.816	0.283	0.666	0.207	0.573	0.844
BR_LF_, ms × mmHg^−1^	11.29 (8.88; 17.65)	9.03 (8.14; 11.14)	16.49 (13.12; 21.97)	25.18 (20.05; 31.65)	**0**.**006**	**0**.**000**	**0**.**000**	**0**.**000**	**0**.**000**	**0**.**017**
BR_HF_, ms × mmHg^−1^	14.85 (10.52; 20.73)	12.00 (7.84; 15.71)	21.54 (16.03; 31.29)	32.03 (23.92; 40.20)	**0**.**008**	**0**.**000**	**0**.**001**	**0**.**000**	**0**.**000**	**0**.**026**

Abbreviations: HR, heart rate; SBP, systolic blood pressure (by Korotkoff); DBP, diastolic blood pressure (by Korotkoff); TP, total power; VLF, very low frequency; LF, low frequency of heart rate variability; HF, high frequency of heart rate variability; SI, stress index; LF_SBP_, low frequency of systolic blood pressure variability (by Penaz); HF_SBP_, high frequency of systolic blood pressure variability (by Penaz); CO, cardiac output; RR, respiratory rate; V_E_, minute lung ventilation; RMSSD, square root of the sum of squares of the differences in the values of consecutive pairs of normal intervals; pNN50, the percentage of NN50 from the total number of consecutive pairs of intervals that differ by more than 50 milliseconds, obtained over the entire time recording; VSI, volume synchronization index; BR_LF_, arterial baroreflex sensitivity in low frequency range; BR_HF_, arterial baroreflex sensitivity in high frequency range.

Bold values are significant differences.

The significant results of the comparison, in our opinion, should also include the lack of differentiation in the state of rest during spontaneous breathing in the indicators of frequently and volume cardiorespiratory interaction—the HI (c.u.) and VSI (dm^3^ × L^−1^), which with different types of HRR did not differ, which proved their sufficient stability in athletes.

### 3.3 Differences in indicators of cardiorespiratory interaction during the breathing maneuver

At the next stage, the main task of the study was to analyze group differences in indicators of cardiorespiratory interaction during tests with controlled breathing (CR). We studied the increments of the specified indicators separately for CR_6_ and CR_15_. As it was mentioned earlier, indicators indicating cardiorespiratory interaction include indicators of frequently (HI) and volume (VSI) synchronization, indicators indicating cardiovascular interaction include indicators of BRS (BR_LF_ and BR_HF_).

#### 3.3.1 Differences in indicators of hildebrandt index during the breathing maneuver

According to the δ HI (c.u.) at CR_6_ and δ HI (c.u.) at CR_15_, central (types I, II) and autonomous (types III, IV) HRV are clearly differentiated (Table 4). That is, the undifferentiated values of the HI during spontaneous breathing in athletes with different types of HRR during controlled breathing CR_6_ and CR_15_ compared to SR clearly determine the central and autonomous variants of HRR. They are respectively characterized by a more significant increase in the HI at CR_6_ and CR_15_ with a predominance of sympathetic influences, as well as a less significant increase in the HI at CR_6_ and unchanged with a tendency to decrease at CR_15_ with a predominance of parasympathetic influences.

**Table 4:** Increments of indicators hildebrandt index (HI) in the examined athletes with type I-IV heart rhythm regulation during the breathing maneuver at CR_6_ and CR_15_ compared to SR, Med (Q_1_; Q_3_).

Parameter		δ SR—CR_6_	δ SR—CR_15_	z	*p*
δHI, c.u.	I	6.67 (5.47; 7.62)	0.71 (−0.16; 1.81)	6.3	0.000
δHI, c.u.	II	6.55 (6.02; 7.37)	0.57 (−0.16; 1.17)	4.7	0.000
δHI, c.u.	III	5.50 (4.31; 6.43)	−0.04 (−1.04; 0.87)	8.0	0.000
δHI, c.u.	IV	5.28 (3.76; 6.16)	−0.16 (−1.81; 0.57)	3.5	0.000
*p*	I–II	0.100	0.999		
*p*	I–III	**0** **.** **000**	**0**.**017**		
*p*	I–IV	**0**.**016**	0.116		
*p*	II–III	**0**.**003**	0.111		
*p*	II–IV	**0**.**027**	0.218		
*p*	III–IV	0.968	0.974		

Abbreviations: HI, hildebrandt index; SR, spontaneous respiration; CR_6_, controlled respiration 0.1 Hz; CR_15,_ controlled respiration 0.25 Hz.

Bold values are significant differences.

The results of the Two Way ANOVA test of the δHI (c.u.) indicator taking into account the type of HRR (Factor B) and controlled breathing (Factor A) are presented in Table 5.

**Table 5:** The results of the Two Way ANOVA test of the δHI (c.u.).

Source	DF	Sum of square (SS)	Mean square (MS)	F statistic (df_1_, df_2_)	*p*-value
Factor A—rows (A)	1	3253.7438	3253.7438	880.7547 (1,358)	**<2**.**2e^−16^**
Factor B—columns (B)	3	141.8665	47.2888	12.8006 (3,358)	**5**.**788e^−8^**
Interaction AB	3	7.2588	2.4196	0.655 (3,358)	0.5803
Error	358	1322.5479	3.6943		
**Total**	**365**	**4725**.**417**	**12**.**9463**		

Abbreviations: A, controlled respiration 6 and 15 times; B, types of heart rhytm regulation (I; II; III; IV).

Bold values are significant differences.

#### 3.3.2 Differences in indicators of volumes synchronization index during the breathing maneuver

There were no significant differences in the *δ*VSI-CR_6_ index (dm^3^ × L^−1^) among athletes with different types of HRR. In one case of comparison, there was a tendency for a difference between the δVSI-CR_15_ (dm^3^ × L^−1^) indices in athletes with types I and II of HRR (Table 6), which indicates a lower reactivity of hemodynamic support of lung ventilation during the development of sympathetic overstrain (type II), which may be an additional criterion for the deterioration of the athletes' condition.

**Table 6:** Increments of indicators volumes synchronization index in the examined athletes with type I–IV heart rhythm regulation during the breathing maneuver at CR_6_ and CR_15_ compared to SR, Med (Q_1_; Q_3_).

Parameter		δ SR—CR_6_	δ SR—CR_15_	z	*p*
δVSI, dm^3^ × L**^−^**^1^	I	−0.157 (−0.246; −0.068)	−0.249 (−0.428; −0.165)	4.6	0.000
δVSI, dm^3^ × L**^−^**^1^	II	−0.106 (−0.227; −0.063)	−0.173 (−0.309; −0.081)	2.6	0.008
δVSI, dm^3^ × L**^−^**^1^	III	−0.120 (−0.207; −0.021)	−0.299 (−0.348; −0.142)	5.8	0.000
δVSI, dm^3^ × L**^−^**^1^	IV	−0.261 (−0.385; −0.088)	−0.349 (−0.463; −0.134)	2.0	0.049
*p*	I–II	0.262	0.051		
*p*	I–III	0.338	0.250		
*p*	I–IV	0.984	0.991		
*p*	II–III	0.932	0.586		
*p*	II–IV	0.332	0.355		
*p*	III–IV	0.466	0.813		

Abbreviations: VSI, volume synchronization index; SR, spontaneous respiration; CR_6_, controlled respiration 0.1 Hz; CR_15_, controlled respiration 0.25 Hz.

The results of the Two Way ANOVA test of the δVSI (dm^3^ × L^−1^) indicator taking into account the type of HRR (Factor B) and controlled breathing (Factor A) are presented in Table 7.

**Table 7:** The results of the Two Way ANOVA test of the δVSI (dm^3^ × L^−1^).

Source	DF	Sum of square (SS)	Mean square (MS)	F statistic (df_1_,df_2_)	*p*-value
Factor A—rows (A)	1	0.8393	0.8393	10.8574 (1,358)	**0**.**001082**
Factor B—columns (B)	3	0.9951	0.3317	4.2909 (3,358)	**0**.**005422**
Interaction AB	3	0.02893	0.009644	0.1248 (3,358)	0.9454
Error	358	27.6752	0.0773		
**Total**	**365**	**29**.**5386**	**0**.**08093**		

Abbreviations: A, controlled respiration 6 and 15 times; B, types of heart rhytm regulation (I; II; III; IV).

Bold values are significant differences.

#### 3.3.3 Differences in indicators of sensitivity of the arterial baroreflex during the breathing maneuver

A number of significant differences during the respiratory maneuver were also obtained among the BRS increments, which indicate interaction in the cardiovascular system.

During spontaneous breathing, the values of BR_LF_ (ms × mmHg^−1^) and BR_HF_ (ms × mmHg^−1^) are quite clearly related to the type of HRR (Table 3). At the same time, the highest BRS values indicate a predominance of parasympathetic influences, and the lowest ones indicate a significant predominance of sympathetic influences. These data confirm the well-known facts regarding the influence of sympathetic and parasympathetic activity on BRS (57).

Considering the data presented in Tables 8, 9, it should be noted that *δ*BR_LF_ and δBR_HF_ at CR_6_ are not differentiated in athletes with types I–III. At the same time, athletes with type IV at CR_6_ differ from other groups in various ways. For δBR_LF_ significantly (*p* = 0.026)—from athletes with type III, with a tendency (*p* = 0.057)—from athletes with type I and do not differ at all from athletes with type II (*p* = 0.141). For δBR_HF_ significantly (*p* = 0.038 and *p* = 0.043)—from athletes with types I and II, respectively, and with a tendency (*p* = 0.071)—from athletes with type III. That is, deep slow breathing, probably causing excessive vagotonic cardiorespiratory control, causes dysregulation in the low-frequency range, which may reflect similar cardiovascular relationships against the background of different initial states during the development of overstrain according to sympathetic (type II) and parasympathetic (type IV) variants. This distinguishes the latter (athletes with type IV) from athletes with moderate parasympathicotonia (type III). On the other hand, changes in baroreflex sensitivity in the high-frequency range (δBR_HF_) at CR_6_ in athletes with type IV still more attest to the features of redundancy characteristic of vagotonia, which is reflected in differences with sympathicotonic variants of HRR, although there is also a certain tendency to differences with moderate parasympathicotonia. On the other hand, hyperventilatory stimulation (CR_15_), which activates the sympathetic link of the ANS and a more pronounced decrease in BRS in the low-frequency and high-frequency ranges (δBR_LF_ and δBR_HF_) in athletes with type IV compared to athletes with types I and II, and with a more pronounced tendency compared to type III. The reduced BRS reactivity (δBR_LF_ and δBR_HF_) in response to a sympathetic stimulus (CR_15_) in athletes with a predominance of sympathetic influences (types I and II) seems to be quite informative.

**Table 8:** Increments of indicators baroreflex sensitivity in low frequency range in the examined athletes with type I-IV heart rhythm regulation during the breathing maneuver at CR_6_ and CR_15_ compared to SR, Med (Q_1_; Q_3_).

Parameter		δ SR—CR_6_	δ SR—CR_15_	z	*p*
δ BR_LF_, ms × mmHg^−1^	I	5.19 (1.31; 8.77)	−3.38 (−8.13; −1.36)	6.3	0.000
δ BR_LF_, ms × mmHg^−1^	II	5.30 (2.88; 10.01)	−2.02 (−5.23; 0.61)	4.7	0.000
δ BR_LF_, ms × mmHg^−1^	III	5.08 (0.11; 11.84)	−5.30 (−10.48; −2.34)	8.0	0.000
δ BR_LF_, ms × mmHg^−1^	IV	1.67 (−6.68; 7.84)	−10.49 (−16.56; −7.12)	3.5	0.000
*p*	I–II	0.995	0.472		
*p*	I–III	0.994	0.536		
*p*	I–IV	0.057	**0** **.** **009**		
*p*	II–III	0.966	**0**.**043**		
*p*	II–IV	0.141	**0**.**001**		
*p*	III–IV	**0**.**026**	0.067		

Abbreviations: BR_LF_, arterial baroreflex sensitivity at LF range; SR, spontaneous respiration; CR_6_, controlled respiration 0.1 Hz; CR_15_, controlled respiration 0.25 Hz.

Bold values are significant differences.

**Table 9:** Increments of indicators baroreflex sensitivity in high frequency range in the examined athletes with type I-IV heart rhythm regulation during the breathing maneuver at CR_6_ and CR_15_ compared to SR, Med (Q_1_; Q_3_).

Parameter		δ SR—CR_6_	δ SR—CR_15_	z	*p*
δ BR_HF_, ms × mmHg^−1^	I	4.30 (−0.82; 10.16)	−6.57 (−9.39; −2.68)	5.7	0.000
δ BR_HF_, ms × mmHg^−1^	II	3.97 (−0.42; 9.08)	−5.30 (−7.16; −1.75)	4.6	0.000
δ BR_HF_, ms × mmHg^−1^	III	3.50 (−5.57; 9.24)	−7.52 (−13.53; −3.32)	7.6	0.000
δ BR_HF_, ms × mmHg^−1^	IV	−3.11 (−12.94; 1.89)	−12.00 (−22.40; −6.17)	3.5	0.000
*p*	I–II	0.995	0.899		
*p*	I–III	0.942	0.472		
*p*	I–IV	**0** **.** **038**	**0**.**000**		
*p*	II–III	0.886	0.221		
*p*	II–IV	**0**.**043**	**0**.**000**		
*p*	III–IV	0.071	**0**.**002**		

Abbreviations: BR_HF_, arterial baroreflex sensitivity at HF range; SR, spontaneous respiration; CR_6_, controlled respiration 0.1 Hz; CR_15_, controlled respiration 0.25 Hz.

Bold values are significant differences.

That is, the differences in BRS increases at CR_6_ and at CR_15_ provide additional criteria for differentiating excessive parasympathetic overstrain in comparison with sympathetic regulation variants and moderate parasympathetic.

The results of the Two Way ANOVA test of the BR_LF_ (ms × mmHg^−1^) indicator taking into account the type of HRR (Factor B) and controlled breathing (Factor A) are presented in Table 10.

**Table 10:** The results of the Two Way ANOVA test of the BR_LF_ (ms × mmHg^−1^).

Source	DF	Sum of Square (SS)	Mean Square (MS)	F Statistic (df,df)	*p*-value
Factor A—rows (A)	1	12466.0695	12466.0695	187.8874 (1,358)	**<2**.**2e**^−^**^16^**
Factor B—columns (B)	3	1321.992	440.664	6.6416 (3,358)	**0**.**0002241**
Interaction AB	3	296.1205	98.7068	1.4877 (3,358)	0.2176
Error	358	23752.8062	66.3486		
**Total**	**365**	**37836**.**9882**	**103**.**663**		

Abbreviations: A, controlled respiration 6 and 15 times; B, types of heart rhytm regulation (I; II; III; IV).

Bold values are significant differences.

The results of the Two Way ANOVA test of the BR_HF_ (ms × mmHg^−1^) indicator taking into account the type of HRR (Factor B) and controlled breathing (Factor A) are presented in Table 11.

**Table 11:** The results of the Two Way ANOVA test of the BR_HF_ (ms × mmHg^−1^).

Source	DF	Sum of square (SS)	Mean square (MS)	F statistic (df_1_, df_2_)	*p*-value
Factor A—rows (A)	1	12432.8258	12432.8258	96.8926 (1,358)	**<2**.**2e^−16^**
Factor B—columns (B)	3	3472.8871	1157.629	9.0217 (3,358)	**0**.**00000897**
Interaction AB	3	69.3075	23.1025	0.18 (3,358)	0.9099
Error	358	45936.9429	128.3155		
**Total**	**365**	**61911**.**9633**	**169**.**6218**		

Abbreviations: A, controlled respiration 6 and 15 times; B, types of heart rhytm regulation (I; II; III; IV).

Bold values are significant differences.

Table 12 summarizes the results obtained from the standpoint of the types of HRR differentiation by absolute values during spontaneous breathing and the increments of cardiorespiratory and cardiovascular interaction indicators during the respiratory maneuver, which may contribute to the algorithmization and supplementation of HRR assessment.

**Table 12:** Differences in the absolute indicators of intersystem interaction and their increments during a breathing maneuver with different types of heart rate regulation.

Types of HRR	I type	II type	III type
II type	SR—BR_LF_ ↓↓; BR_HF_ ↓↓ CR_15—_δVSI↓=		SR—BR_LF_ ↓↓↓; BR_HF_ ↓↓↓ CR_6_—δHI ↑↑ CR_15—_δBR_LF_ ↑
III type	SR—BR_LF_ ↑↑↑; BR_HF_ ↑↑↑ CR_6—_δHI ↓↓↓ CR_15—_δHI ↓	SR—BR_LF_ ↑↑↑; BR_HF_ ↑↑↑ CR_6—_δHI ↓↓ CR_15—_δBR_LF_ ↓	
IV type	SR—BR_LF_ ↑↑↑; BR_HF_ ↑↑↑ CR_6—_δHI ↓; δBR_LF_ ↓=; δBR_HF_ ↓ CR_15—_δBR_LF_ ↓↓; δBR_HF_ ↓↓↓	SR—BR_LF_ ↑↑↑; BR_HF_ ↑↑↑ CR_6—_δHI ↓↓; δBR_HF_ ↓ CR_15—_δBR_LF_ ↓↓; δBR_HF_ ↓↓	SR—BR_LF_ ↑; BR_HF_ ↑ CR_6—_δBR_LF_ ↓; δBR_HF_↓= CR_15—_δBR_LF_ ↓=; δBR_HF_ ↓↓

Abbreviations: See above.

Note: ↑ or ↓—more or less, *p* < 0.05; ↑↑ or ↓↓—more or less, *p* < 0.01; ↑↑↑ or ↓↓↓—more or less, *p* < 0.001; ↑= or ↓=—tendence to more or less.

Moderate and severe sympathicotonia (types I and II) are poorly differentiated by the indicators of cardiorespiratory interaction during controlled breathing. The only indicator indicating a tendency to differences is the δVSI indicator, which during hyperventilation in athletes with an excessive predominance of sympathetic influences (type II) is lower compared to athletes with moderate sympathicotonia (type I). However, this may indicate dysregulation, which determines the relative deterioration of the heart pumping function during sympathetic activation during CR_15_.

Moderate and severe parasympathicotonia (types III and IV) are not differentiated at all by the indicators of frequency (HI) and volume (VSI) cardiorespiratory synchronization during controlled breathing. However, significant are the differences in vagotonic stimulation (CR_6_) by the indicator—δBR_LF_ and sympathicotonic stimulation (CR_15_) by the indicator *δ*BR_HF_, the increase of which in parasympathetic overstrain (IV type) indicates a significant decrease in BRS during controlled breathing.

The differences in the increase in the HI indicator during CR_6_ are noteworthy, which differentiate all variants of sympathicotonic and parasympathicotonic states from each other due to the different increase in HR (min^−1^), which in different types increases to different extents when performing vagostimulating breathing (CR_6_). During CR_15_, the increase in this indicator allows you to differentiate moderate parasympathicotonia (III type) from moderate sympathicotonia (I type).

## 4 Discussion

In the practice of sports medicine, to assess the functional state of the athlete's body as a whole and the activity of the cardiovascular system during current examinations in the training process is most often used the analysis of HRV indicators, which reflect the degree of load on the autonomous regulation of the cardiovascular system (31, 86–88). However, their joint analysis with the parameters of changes in vascular tone and spontaneous breathing has not been sufficiently studied (40, 89–92). In previous studies of changes in HRV at rest during the training process, its significant dependence on the frequency of spontaneous breathing and tidal volume was shown (62). This prompted us to use controlled breathing tests to standardize and unify the assessment of HRV changes (64, 67, 93). A breathing maneuver procedure has been proposed that allows us to detect the body's reactivity to influences that stimulate, at least, the activation of the sympathetic and parasympathetic branches of the ANS (69). It is important during testing to focus on the choice of frequency and duration of breathing phases. The CR_6_ with a fixed duration of inhalation and exhalation (5 s each) has been studied in many works and is considered resonant (0.1 Hz) for activating vagal baroreflex effects on HR (94, 95). This allows us to investigate the mechanism of baroreflex activation from the standpoint of its presence and severity in standardized conditions. After all, baroreflex activation also occurs during SR, but depends on its frequency and duration of inhalation and exhalation phases. Regarding the CR_15_ with a fixed duration of inhalation and exhalation (2 s each), something should be explained. The breathing frequency itself is within the optimal values for a healthy person. However, the duration of the inhalation and exhalation phases is significantly different from the proper ones (1:2). This also causes a change in the wave characteristics of the HRV (68), but with a constant frequency component (0.25 Hz), which is reflected in the HRV indicators, in which there is a more rigid redistribution towards the HF component compared to SR which is a physiological (67). With this breathing option, a certain tension in gas exchange occurs. As a rule, to implement such a breathing rhythm, a person must exhale more actively. As a result, the next breath will also be strengthened, but within the duration (2 s), which in turn will contribute to an increase in the tidal volume. This leads to moderate hyperventilation, which can reach 20 or more (L × min^−1^) at rest (69). The main thing in such conditions is the ability of the exhalation muscles to releasing the lungs from air. Accordingly, there is an impact on the circulatory system, namely the possibility of implementing the baroreflex mechanism, the sensitivity of which in this case is significantly reduced. That is, breathing with a controlled rhythm (2:2) is not physiological and allows us to characterize the response to moderate hyperventilation.

The main indicators that determined the differences between the types of HRR were the BRS indicators (BR_LF_ and BR_HF_) during SR, which characterize the functioning of the negative feedback system that buffers short-term fluctuations in blood pressure by modifying cardiovascular parameters—HR (min^−1^) and SV (ml). There are a sufficient number of publications that analyze changes in BRS under the influence of various factors, including training loads, which indicate the features of intersystem interaction and allow us to characterize the level of stress of the body after performing the load, its recovery, or the tendency to develop overstrain of the cardiovascular system (94, 96, 97). At the same time, the clinical significance of a decrease in BRS during SR is determined by the ability to early diagnose disorders of autonomic function (98–100) due to a decrease in inhibitory activity and an imbalance of physiological sympathovagal outflow to the heart, which leads to chronic adrenergic activation (101). The effects of a decrease in BRS are usually associated with a decrease in the LF component of HRV, which was confirmed in this work. Against the background of an increase in HR (min^−1^), the LF component of HRV (ms^2^) decreases and the LF component of BP (mmHg^2^) increases (102), which additionally affects the decrease in the BR_LF_ (ms × mm Hg^−1^). The results obtained during the study of highly qualified athletes with signs of the development of sympathetic and parasympathetic overstrain under the influence of intense physical load also showed that BR_LF_ (ms × mmHg^−1^) and BR_HF_ (ms × mmHg^−1^) during SR in athletes with both types of overstrain decrease immediately after exercise, and do not recover to their original values the next day (40, 103). A study of athletes at competitions showed differences in the recovery of BR_LF_ (ms × mmHg^−1^) and BR_HF_ (ms × mm Hg^−1^). Guzii and Romanchuk (102), which may be an additional criterion for the body's recovery after training and competitions. Subsequently, it was shown that conditions with impaired bronchial patency and hypertension also significantly affect baroreceptor function, which may underlie the mechanisms of formation of hypertensive states (65, 66, 104), and may also be useful for determining and predicting changes in asthma formation due to physical stress and the development of parasympathetic dysregulation. In general, BRS indicators (BR_LF_ and BR_HF_) are key in determining the mechanisms of maintaining vascular homeostasis and indicators of autonomic control (70, 105, 106). The results obtained in this study indicate that BRS in SR is associated with the features of autonomic HRV and may indicate a significant predominance of central or autonomic mechanisms and evidence the development of dysregulation. The lowest BRS is during the development of fatigue, the formation of sympathetic overstrain (type II), and the highest is during the formation of supercompensation states, or parasympathetic overstrain (type IV). Previous studies have shown that BRS significantly increases during deep slow breathing (CR_6_) from 14.2 (9.7; 20.1) to 20.4 (14.6; 26.7), *p* = 0.000 for BR_LF_ (ms × mmHg^−1^) and from 18.3 (11.9; 27.5) to 20.8 (15.4; 30.4), *p* = 0.000 for BR_HF_ (ms × mmHg^−1^). This was confirmed by the results of dynamic observation of endurance athletes, who showed a more pronounced increase in BR_LF_ (ms × mmHg^−1^) at CR_6_ (107), which may also indicate the formation of excessive control over cardiorespiratory relationships (45). At the same time, with deeper, more frequent controlled breathing (CR_15_), BRS significantly decreases, even compared to SR, to 9.4 (6.8; 12.9), *p* = 0.000 for BR_LF_ (ms × mm Hg^−1^) and to 10.3 (6.2; 15.6), *p* = 0.000 for BR_HF_ (ms × mmHg^−1^) (69), which is due to the activation of sympathetic influences during moderate hyperventilation. In general, the results obtained in this study confirm and complement the known data on the effect of controlled breathing on BRS (95, 108–110). The results of this study demonstrate that δBR_LF_ at CR_6_ in athletes with type II and IV, which are similar, suggest the formation of dysregulations by sympathetic and parasympathetic types against the background of excessive cardiorespiratory control during vagal-stimulating breathing. At the same time, during CR_15_, δBR_HF_ in athletes of these groups is significantly different, indicating different reactivity to sympathostimulating influences.

A well-known and widely used indicator reflecting cardiorespiratory interaction is the Hildebrandt index (72). The possibilities of simultaneous measurement of HR (min^−1^) and RR (min^−1^) significantly complement the known data, taking into account many components that affect the value of this indicator. Currently, technologies and clothing devices are being developed that will allow for more accurate determination and clear analysis of this parameter in different conditions (111). The technology of simultaneous measurement of indicators of the cardiovascular and respiratory systems makes it possible to develop a number of both frequency and discrete parameters of cardiorespiratory interaction (73, 85, 90). As an example, we can cite the data obtained directly in the procedure of manual correction of the thoracic spine, when this indicator changed due to significant changes in the duration of exhalation (112). In this study, it was shown that none of the groups that differed in the type of HRR during SR differed in HI (cu), despite the differences in HR (min^−1^). On the other hand, when performing the respiratory maneuver, differences are recorded that clearly distinguish the central and autonomous types of HRR. It was previously shown that the HI (cu) in the group of active men during tests with controlled breathing significantly increases from 4.77 (3.98; 6.20) to 10.92 (9.97; 12.14), *p* = 0.000 at CR_6_, but remains unchanged compared to SR 5.00 (4.48; 5.84), *p* = 0.209 at CR_15_ (69). Other authors have demonstrated its increase during physical activity, which occurred due to a relative increase in HR (min^−1^) compared to RR (min^−1^). The latter, in their opinion, indicates its informativeness regarding the strengthening of sympathetic influences and the “physiological price” of the work performed and predicts the refusal of intensive physical activity (113). Here it is worth recalling the results of the analysis of the breathing pattern, which was carried out in athletes with different types of HRR and showed the development of symptoms of expiratory insufficiency in athletes with type II (84). From these positions, a less pronounced increase in HI (cu) when performing a breathing maneuver with CR_6_ and CR_15_ at the end of the training cycle for the development of strength endurance (107) indicates an increase in the reserve capabilities of the cardiorespiratory system.

The possibility of simultaneous registration of ECG and respiratory air flows allowed us to verify the use of a discrete parameter (VSI, dm^3^ × L^−1^), which characterizes the ratio between cardiac output and minute lungs ventilation. It takes into account adaptive changes in both the cardiovascular and respiratory systems, which are somehow interconnected, due to the intake and transport of oxygen in the body. When studying VSI (dm^3^ × L^−1^), which characterizes volumetric synchronization, it was previously shown that when performing a respiratory maneuver with a change in RR (min^−1^), it significantly decreases from 0.586 (0.477; 0.745) during SR to 0.441 (0.327; 0.582) at CR_6_ to 0.360 (0.247; 0.477), *p* = 0.000 at CR_15_ (69). Other studies have shown that it increases with load (113). That is, this indicator can be used to characterize the adequacy of the response to physical activity and energy expenditure. When examining 202 highly qualified athletes before, after and the next morning after training, fairly stable indicators were obtained, which indicates the possibility of using this indicator to characterize the individual ability of the body to tolerate the load and recover after it—before 0.566 (0.448; 0.765), after 0.574 (0.432; 0.790), the next morning 0.593 (0.482; 0.891) (114). At the same time, the results obtained during the respiratory maneuver indicate the peculiarities of its changes under the influence of training on the development of strength endurance, which indicates the adaptation of cardiorespiratory relationships under the influence of training, which is characterized by efficiency and economy oxygen supply, which prevents the development of excessive sympathoadrenal activation. Thus, strength endurance training leads to an increase and VSI index (dm^3^ × L^−1^) during SR at rest from 0.597 (0.490; 0.832) to 0.725 (0.564; 1.148), *p* = 0.008, which reflects its increase during CR_6_ from 0.327 (0.382; 0.529) to 0.532 (0.441; 0.723), *p* = 0.012, and at CR_15_ from 0.245 (0.339; 0.455) to 0.481 (0.373; 0.616), *p* = 0.003. This proved better adaptation of the cardiorespiratory system to the suppression of postganglionic activity at CR_6_ and the activation of hyperventilation at CR_15_. From these points of view, the results of this study allowed us to clearly differentiate sympathetic overstrain (type II), which was characterized by the lowest variability of the VSI indicator (dm^3^ × L^−1^) during the respiratory maneuver, which can be used as a separate parameter characterizing the development of functional or non-functional overstrain of the athletes' body (52, 115).

That is, determining changes in intersystem relationships during the performance of a respiratory maneuver with a change in RR in athletes with different types of HRR made it possible to supplement information about the conditions for the occurrence of excessive predominance of sympathetic and parasympathetic HRR. The latter characterize the development of sympathetic and parasympathetic overstrain, which correlates with the development of functional and non-functional overstrain and precedes the formation of overtraining in the body of athletes. That's may contribute to the detection of these conditions in the field examinations of athletes during current and operational control.

## 5 Limitation

The study analyzed the results of testing highly qualified athletes of different sports at different periods of the training process, which does not allow linking the results obtained with the type of loads performed and direct VO2peak. It is also necessary to emphasize that the examined group of athletes was engaged in sports of different orientation and intensity. This somewhat limits the possibility of taking into account indicators of intersystem interaction in determining the functional state of the body in athletes of specific sports. Also, the issues of group differentiation due to their uneven distribution in this study have not been fully resolved. At the same time, the expressness of this study (6 min), taking into account the multifunctionality of such an examination and the possibility of conducting it in field conditions, allows not only to unify and individualize the multiparameter assessment, but also to refine it in the future, which opens up new prospects for current monitoring of the athletes’ condition.

## 6 Conclusions

The use of a respiratory maneuver in a combined study of the cardiorespiratory system in the conditions of current control of athletes showed informativeness in the differentiation of HRR types and states of functional overstrain.

## Data Availability

The datasets presented in this study can be found in online repositories. The names of the repository/repositories and accession number(s) can be found in the article/Supplementary Material.
