# Supplementary material for: Peculiarities of cardio-respiratory relationships in qualified athletes with different types of heart rhythm regulation according to respiratory maneuver data
Source: Front Sports Act Living. 2025 Jan 13;6:1451643. doi: 10.3389/fspor.2024.1451643 (PMC11769980; doi:10.3389/fspor.2024.1451643)
Supplement: Supplementary file 1 [file Datasheet1.pdf]

Two sample ANOVA - fixed-test, using F distribution (right-tailed) [δVSI, $dm^3 \times L^{-1}$]

(Created with use resurses of www.statskingdom.com)

Factor – A (CR_6; CR_{15})

1. H_0 hypothesis

Since the p-value < α, H_0 is rejected.

Some of the groups' averages consider to be not equal.

In other words, the sample difference between the averages of some groups is big enough to be statistically significant.

2. P-value

The p-value equals 0.001082, (P(x≤10.8574) = 0.9989). It means that the chance of type I error (rejecting a correct H_0) is small: 0.001082 (0.11%). The smaller the p-value the more it supports H_1.

3. Test statistic

The test statistic F equals 10.8574, which is not in the 95% region of acceptance: [0, 3.8676].

4. Effect size

The observed effect size η^2 is small, 0.029. This indicates that the magnitude of the difference between the averages is small.

Factor – B (type; I; II; III; IV)

1. H_0 hypothesis

Since the p-value < α, H_0 is rejected.

Some of the groups' averages consider to be not equal.

In other words, the sample difference between the averages of some groups is big enough to be statistically significant.

2. P-value

The p-value equals 0.005422, (P(x≤4.2909) = 0.9946). It means that the chance of type I error (rejecting a correct H_0) is small: 0.005422 (0.54%). The smaller the p-value the more it supports H_1.

3. Test statistic

The test statistic F equals 4.2909, which is not in the 95% region of acceptance: [0, 2.6298].

4. Effect size

The observed effect size η^2 is small, 0.035. This indicates that the magnitude of the difference between the averages is small.

Interaction AB

1. H hypothesis

Since the p-value > α, H_0 can not be rejected.

The averages of all groups assume to be equal.

In other words, the difference between the sample averages of all groups is not big enough to be statistically significant.

A non-significance result can not prove that H_0 is correct, only that the null assumption can not be rejected.

2. P-value

The p-value equals 0.9454, (P(x≤0.1248) = 0.05456). It means that the chance of type I error, rejecting a correct H_0 , is too high: 0.9454 (94.54%). The larger the pvalue the more it supports H_0.

3. Test statistic

The test statistic F_{AB} equals 0.1248, which is in the 95% region of acceptance: [0, 2.6298].

4. Effect size

The observed effect size η^2 is very small, 0.001. This indicates that the magnitude of the difference between the averages is very small.

Source	DF	Sum of Square (SS)	Mean Square (MS)	F Statistic (df_1,df_2)	P-value
Factor A - rows (A)	1	0.8393	0.8393	10.8574 (1,358)	**0.001082**
Factor B - columns (B)	3	0.9951	0.3317	4.2909 (3,358)	**0.005422**
Interaction AB	3	0.02893	0.009644	0.1248 (3,358)	0.9454
Error	358	27.6752	0.0773		
**Total**	**365**	**29.5386**	**0.08093**		

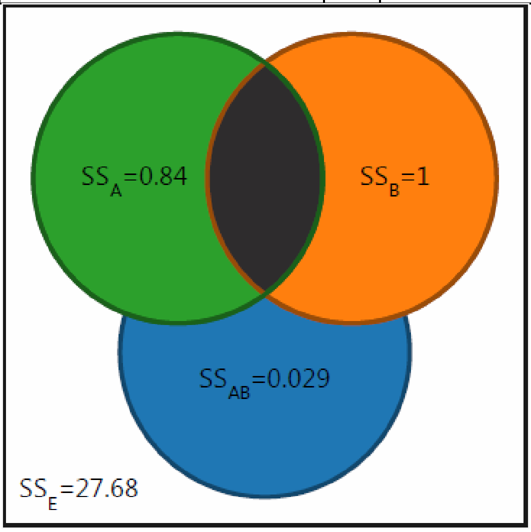

SS_T=29.54 SS_M=29.54 SS_{OVL} = 0

The unbalanced design induces correlations between the factors, with positive correlations leading to positive overlap and negative correlations resulting in negative overlap. Since the overlapping variation between A and B (SS) is positive, this overlapping variation reduces the sum of squares for both factor A and factor B, and the plot displays it in black.

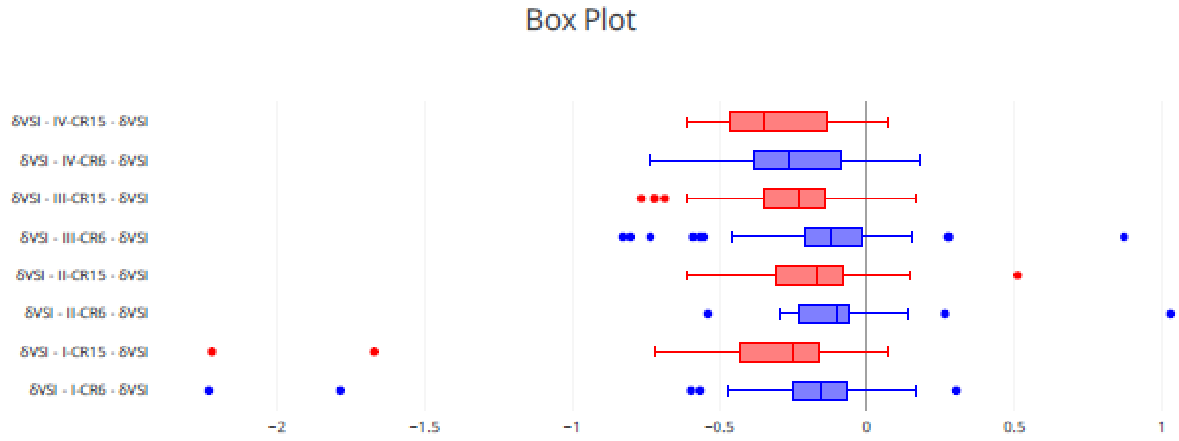

⦿ Type

Most likely there an interaction effect doesn't exist, or only a very small interaction exists.
Even if there is no interaction there may still be a correlation between the two factors unless the cell sizes represents the population proportions, and usually this is not exactly the case.

⦿ Outliers

Outliers' detection method: Tukey Fence, k=1.5.
The residuals contains 15 potential outliers, which is **4.1%** of the observations. (-2.231, -1.785, 0.302, -2.221, -1.672, 1.028, 0.51, 0.871, -0.828, -0.735, -0.803, -0.723, -0.718, -0.767, -0.738).
The two way ANOVA-test is robust to the presence of outliers.

⦿ Normality

The assumption was checked based on the Shapiro-Wilk Test. (α=0.05)
It is assumed that the residuals does not follow the normal distribution (p-value is 0).
The test is considered robust for moderate violation of the normality assumption.
The sample size is greater than 30, hence if the sample data is reasonably symmetric, the statistic may distribute approximately normally (central limit theorem)
You should check if the sample data is reasonably symmetric around the average.
If not, you should check the data transformation, e.g. log transformation and square-root transformation.
If none of the transformations work, you should run a non-parametric test.
The relevant non-parametric test is the Wilcoxon signed ranks test.

⦿ Test power: Factor - A

The test priori power is strong 1

⦿ Test power: Factor - B

The test priori power is strong 1

⦿ Test power: Interaction

The test priori power is strong 1

Two sample ANOVA - fixed-test, using F distribution (right-tailed) [δHI, c.u.]

Factor – A (CR_6; CR_{15})

1. H_0 hypothesis

Since the p-value < α, H_0 is rejected.
Some of the groups' averages consider to be not equal.
In other words, the sample difference between the averages of some groups is big enough to be statistically significant.

2. P-value

The p-value equals 0, (P(x≤880.7547) = 1). It means that the chance of type I error (rejecting a correct H_0) is small: 0 (0%). The smaller the p-value the more it supports H_1.

3. Test statistic

The test statistic F_A equals 880.7547, which is not in the 95% region of acceptance: [0, 3.8676].

4. Effect size

The observed effect size η^2 is large, 0.71. This indicates that the magnitude of the difference between the averages is large.

Factor – B (type; I; II; III; IV)

1. H_0 hypothesis

Since the p-value < α, H_0 is rejected.
Some of the groups' averages consider to be not equal.
In other words, the sample difference between the averages of some groups is big enough to be statistically significant.

2. P-value

The p-value equals $5.788e^{-8}$, (P(x≤12.8006) = 1). It means that the chance of type I error (rejecting a correct H_0) is small: $5.788e^{-8}$ (0.0000058%). The smaller the p-value the more it supports H_1.

3. Test statistic

The test statistic F_B equals 12.8006, which is not in the 95% region of acceptance: [0, 2.6298].

4. Effect size

The observed effect size η^2 is medium, 0.097. This indicates that the magnitude of the difference between the averages is medium.

Interaction AB

1. H_0 hypothesis

Since the p-value > α, H_0 can not be rejected.
The averages of all groups assume to be equal.
In other words, the difference between the sample averages of all groups is not big enough to be statistically significant.
A non-significance result can not prove that H_0 is correct, only that the null assumption can not be rejected.

2. P-value

The p-value equals 0.5803, ($P(x \le 0.655) = 0.4197$). It means that the chance of type I error, rejecting a correct H_0, is too high: 0.5803 (58.03%). The larger the p value the more it supports H_0.

3. Test statistic

The test statistic F_{AB} equals 0.655, which is in the 95% region of acceptance: [0, 2.6298].

4. Effect size

The observed effect size η^2 is very small, 0.0055. This indicates that the magnitude of the difference between the averages is very small.

Source	DF	Sum of Square (SS)	Mean Square (MS)	F Statistic (df_1,df_2)	P-value
Factor A - rows (A)	1	3253.7438	3253.7438	880.7547 (1,358)	**< $2.2e^{-16}$**
Factor B - columns (B)	3	141.8665	47.2888	12.8006 (3,358)	**$5.788e^{-8}$**
Interaction AB	3	7.2588	2.4196	0.655 (3,358)	0.5803
Error	358	1322.5479	3.6943		
**Total**	**365**	**4725.417**	**12.9463**		

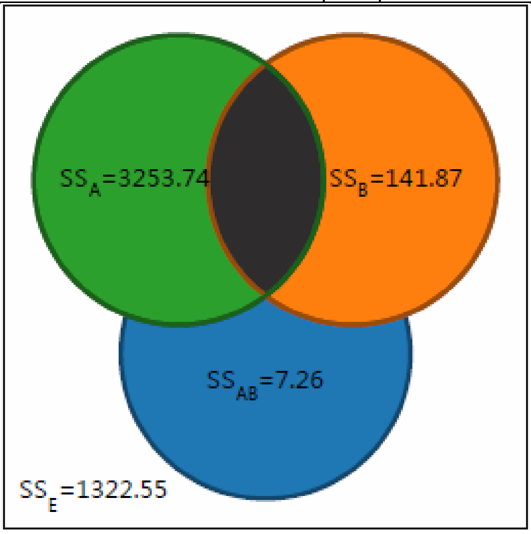

SS_T=4725.42 SS_M=4725.42 SS_{OVL} = 0

The unbalanced design induces correlations between the factors, with positive correlations leading to positive overlap and negative correlations resulting in negative overlap. Since the overlapping variation between A and B (SS) is positive, this overlapping variation reduces the sum of squares for both factor A and factor B, and the plot displays it in black.

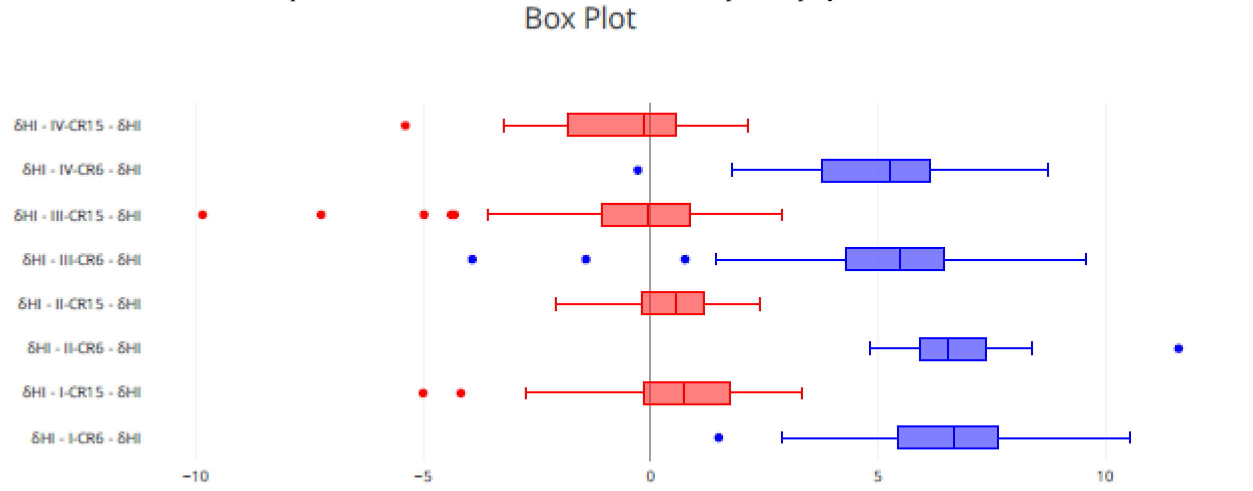

⦿ Type

Most likely there an interaction effect doesn't exist, or only a very small interaction exists.

Even if there is no interaction there may still be a correlation between the two factors unless the cell sizes represents the population proportions, and usually this is not exactly the case.

◉ Outliers

Outliers' detection method: Tukey Fence, k=1.5.

The residuals contains 13 potential outliers, which is **3.55%** of the observations. (1.5, -5, -4.17, 11.62, 0.76, -3.92, -1.42, 9.58, -4.98, -9.85, -7.24, -0.28, -5.39).

The two way ANOVA-test is robust to the presence of outliers.

◉ Normality

The assumption was checked based on the Shapiro-Wilk Test. (α=0.05)

It is assumed that the residuals does not follow the normal distribution (p-value is 1.229e-10).

The test is considered robust for moderate violation of the normality assumption.

The sample size is greater than 30, hence if the sample data is reasonably symmetric, the statistic may distribute approximately normally (central limit theorem)

You should check if the sample data is reasonably symmetric around the average.

If not, you should check the data transformation, e.g. log transformation and square-root transformation.

If none of the transformations work, you should run a non-parametric test.

The relevant non-parametric test is the Wilcoxon signed ranks test.

◉ Test power: Factor - A

The test priori power is strong 1

◉ Test power: Factor - B

The test priori power is strong 1

◉ Test power: Interaction

The test priori power is strong 1

Two sample ANOVA - fixed-test, using F distribution (right-tailed) [δ BR$_{LF}$, ms×mmHg^{-1}]

Factor – A (CR$_6$; CR$_{15}$)

1. H_0 hypothesis

Since the p-value < α, H_0 is rejected.

Some of the groups' averages consider to be not equal.

In other words, the sample difference between the averages of some groups is big enough to be statistically significant.

2. P-value

The p-value equals 0, ($P(x\leq187.8874) = 1$). It means that the chance of type I error (rejecting a correct H_0) is small: 0 (0%). The smaller the p-value the more it supports H_1.

3. Test statistic

The test statistic F_A equals 187.8874, which is not in the 95% region of acceptance: [0, 3.8676].

4. Effect size

The observed effect size η^2 is large, 0.34. This indicates that the magnitude of the difference between the averages is large.

Factor – B (type; I; II; III; IV)

1. H_0 hypothesis

Since the p-value < α, H is rejected.

Some of the groups' averages consider to be not equal.

In other words, the sample difference between the averages of some groups is big enough to be statistically significant.

2. P-value

The p-value equals 0.0002241, ($P(x\leq6.6416) = 0.9998$). It means that the chance of type I error (rejecting a correct H_0) is small: 0.0002241 (0.022%). The smaller the p-value the more it supports H_1.

3. Test statistic

The test statistic F_B equals 6.6416, which is not in the 95% region of acceptance: [0, 2.6298].

4. Effect size

The observed effect size η^2 is small, 0.053. This indicates that the magnitude of the difference between the averages is small.

Interaction AB

1. H_0 hypothesis

Since the p-value > α, H_0 can not be rejected.

The averages of all groups assume to be equal.

In other words, the difference between the sample averages of all groups is not big enough to be statistically significant.

A non-significance result can not prove that H_0 is correct, only that the null assumption can not be rejected.

2. P-value

The p-value equals 0.2176, ($P(x\leq1.4877) = 0.7824$). It means that the chance of type I error, rejecting a correct H_0, is too high: 0.2176 (21.76%). The larger the p value the more it supports H_0.

3. Test statistic

The test statistic F_{AB} equals 1.4877, which is in the 95% region of acceptance: [0, 2.6298].

4. Effect size

The observed effect size η^2 is small, 0.012. This indicates that the magnitude of the difference between the averages is small.

Source	DF	Sum of Square (SS)	Mean Square (MS)	F Statistic (df,df)	P-value
Factor A - rows (A)	1	12466.0695	12466.0695	187.8874 (1,358)	**< 2.2e^{-16}**
Factor B - columns (B)	3	1321.992	440.664	6.6416 (3,358)	**0.0002241**
Interaction AB	3	296.1205	98.7068	1.4877 (3,358)	0.2176
Error	358	23752.8062	66.3486		
**Total**	**365**	**37836.9882**	**103.663**		

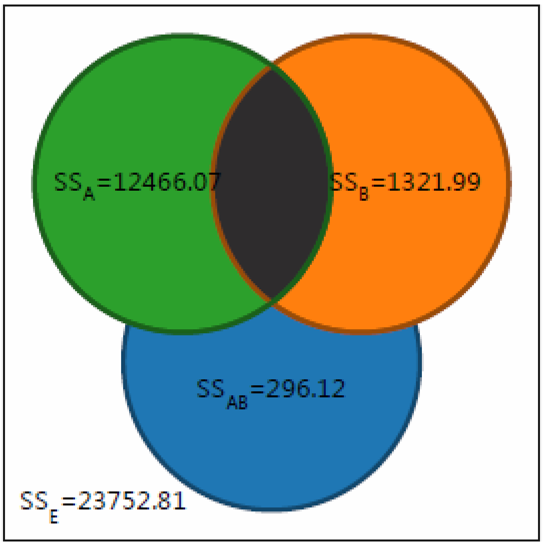

SS_T=37836.99 SS_M=37836.99 SS_{OVL} = 2.2e-11

The unbalanced design induces correlations between the factors, with positive correlations leading to positive overlap and negative correlations resulting in negative overlap. Since the overlapping variation between A and B (SS) is positive, this overlapping variation reduces the sum of squares for both factor A and factor B, and the plot displays it in black.

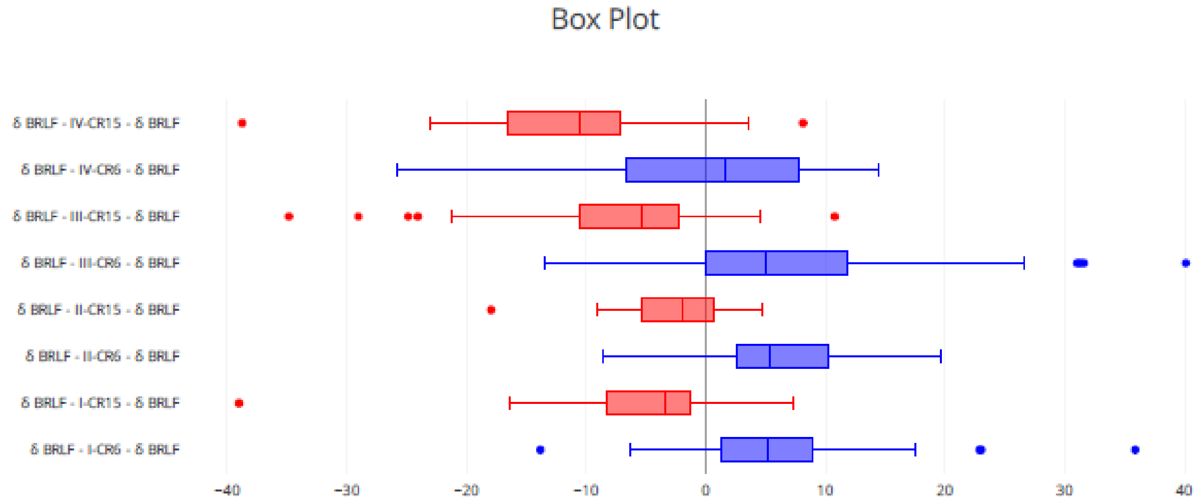

Type

Most likely there an interaction effect doesn't exist, or only a very small interaction exists. Even if there is no interaction there may still be a correlation between the two factors unless the cell sizes represents the population proportions, and usually this is not exactly the case.

Outliers

Outliers' detection method: Tukey Fence, k=1.5. The residuals contains 19 potential outliers, which is **5.19%** of the observations. (35.9, -13.83, -39.03, -13.41, 26.54, 40.13, 31.05, 25.42, 31.59, -11.23, 31.28, 10.78, -24.89, -29.05, -24.09, -34.86, -25.84, -38.77, 8.13). The **two way ANOVA**-test is robust to the presence of outliers.

Normality

The assumption was checked based on the Shapiro-Wilk Test. (α=0.05) It is assumed that **the residuals does not** follow the normal distribution (p-value is $2.662e^{-8}$). The test is considered robust for moderate violation of the normality assumption. The sample size is greater than 30, hence if the sample data is reasonably symmetric, the statistic may distribute approximately normally (central limit theorem) You should check if the sample data is reasonably symmetric around the average. If not, you should check the data transformation, e.g. log transformation and square-root transformation. If none of the transformations work, you should run a non-parametric test. The relevant non-parametric test is the Wilcoxon signed ranks test.

Test power: Factor - A

The test priori power is strong **1**

Test power: Factor - B

The test priori power is strong **1**

Test power: Interaction

The test priori power is strong **1**

Two sample ANOVA - fixed-test, using F distribution (right-tailed) [δBR_{HF}, ms×mmHg^{-1}]

Factor – A (CR_6; CR_{15})

1. H_0 hypothesis

Since the p-value < α, H_0 is rejected.

Some of the groups' averages consider to be not equal.

In other words, the sample difference between the averages of some groups is big enough to be statistically significant.
2. P-value
The p-value equals 0, ($P(x \le 96.8926) = 1$). It means that the chance of type I error (rejecting a correct H_0) is small: 0 (0%). The smaller the p-value the more it supports H_1.
3. Test statistic
The test statistic F_A equals 96.8926, which is not in the 95% region of acceptance: [0, 3.8676].
4. Effect size
The observed effect size η^2 is large, 0.21. This indicates that the magnitude of the difference between the averages is large.
Factor – B (type; I; II; III; IV)
1. H_0 hypothesis
Since the p-value < α, H_0 is rejected.
Some of the groups' averages consider to be not equal.
In other words, the sample difference between the averages of some groups is big enough to be statistically significant.
2. P-value
The p-value equals 0.00000897, ($P(x \le 9.0217) = 1$). It means that the chance of type I error (rejecting a correct H_0) is small: 0.00000897 (0.0009%). The smaller the p-value the more it supports H_1.
3. Test statistic
The test statistic F_B equals 9.0217, which is not in the 95% region of acceptance: [0, 2.6298].
4. Effect size
The observed effect size η^2 is medium, 0.07. This indicates that the magnitude of the difference between the averages is medium.
Interaction AB
1. H hypothesis
Since the p-value > α, H_0 can not be rejected.
The averages of all groups assume to be equal.
In other words, the difference between the sample averages of all groups is not big enough to be statistically significant.
A non-significance result can not prove that H_0 is correct, only that the null assumption can not be rejected.
2. P-value
The p-value equals 0.9099, ($P(x \le 0.18) = 0.09009$). It means that the chance of type I error, rejecting a correct H_0, is too high: 0.9099 (90.99%). The larger the p value the more it supports H_0.
3. Test statistic
The test statistic F_{AB} equals 0.18, which is in the 95% region of acceptance: [0, 2.6298].
4. Effect size
The observed effect size η^2 is very small, 0.0015. This indicates that the magnitude of the difference between the averages is very small.

Source	DF	Sum of Square (SS)	Mean Square (MS)	F Statistic (df_1,df_2)	P-value
Factor A - rows (A)	1	12432.8258	12432.8258	96.8926 (1,358)	**< $2.2e^{-16}$**
Factor B - columns (B)	3	3472.8871	1157.629	9.0217 (3,358)	**0.00000897**
Interaction AB	3	69.3075	23.1025	0.18 (3,358)	0.9099
Error	358	45936.9429	128.3155		
**Total**	**365**	**61911.9633**	**169.6218**		

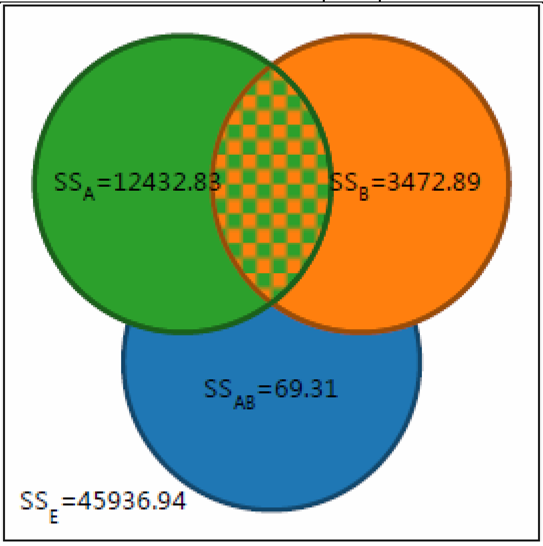

SS_T=61911.96 SS_M=61911.96 SS_{OVL} = $-4.4e^{-11}$
The unbalanced design induces correlations between the factors, with positive correlations leading to positive overlap and negative correlations resulting in negative overlap. Usually, the overlapping variation between A and B (SS) is positive, but in this case, it is negative, representing double-counted variation (referred to as 'underlapping'). This negative overlap increases the sum of squares for both factor A and factor B, resulting in a checkered pattern in the plot. In this case, the model sum of squares (SS) is larger than the total sum of squares (SS).

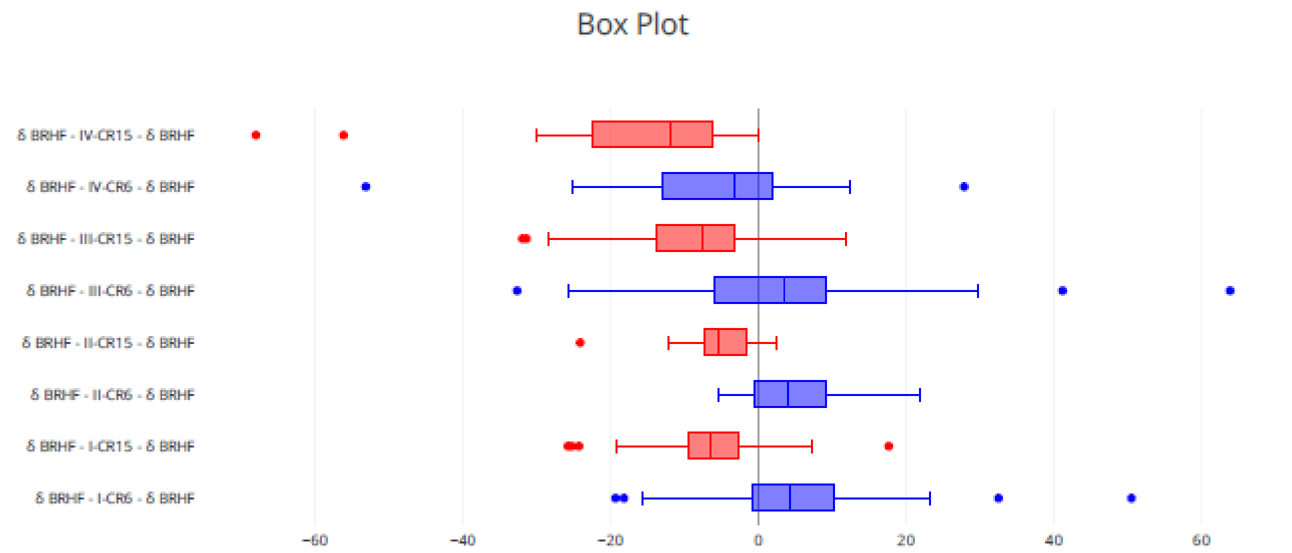

Type

Most likely there an interaction effect doesn't exist, or only a very small interaction exists.
Even if there is no interaction there may still be a correlation between the two factors unless the cell sizes represents the population proportions, and usually this is not exactly the case.

◉ Outliers

Outliers' detection method: Tukey Fence, k=1.5.
The residuals contains 24 potential outliers, which is **6.56%** of the observations. (50.53, 32.5, -15.61, -19.32, -18.19, 17.67, -19.67, -17.88, -25.73, -20.96, 63.87, 29.82, 41.17, -32.61, 23.58, -28.43, 11.81, -31.92, -31.35, -25.09, 27.86, -53.13, -56.13, -67.98).
The two way ANOVA-test is robust to the presence of outliers.

◉ Normality

The assumption was checked based on the Shapiro-Wilk Test. (α=0.05)
It is assumed that the residuals does not follow the normal distribution (p-value is 7.407e-12).
The test is considered robust for moderate violation of the normality assumption.
The sample size is greater than 30, hence if the sample data is reasonably symmetric, the statistic may distribute approximately normally (central limit theorem)
You should check if the sample data is reasonably symmetric around the average.
If not, you should check the data transformation, e.g. log transformation and square-root transformation.
If none of the transformations work, you should run a non-parametric test.
The relevant non-parametric test is the Wilcoxon signed ranks test.

◉ Test power: Factor - A

The test priori power is strong 1

◉ Test power: Factor - B

The test priori power is strong 1

◉ Test power: Interaction

The test priori power is strong 1
